# A randomized controlled trial for acupuncture combined with conventional therapy in the treatment of pain caused by prostate cancer: Study protocol clinical trial (SPIRIT compliant)

**DOI:** 10.1097/MD.0000000000019609

**Published:** 2020-04-03

**Authors:** Yi Lei, Yunyun Duan, Jisheng Wang, Xudong Yu, Sheng Deng, Ruijia Liu, Hongmei Si, Jiameng Li, Bao Zhang

**Affiliations:** a The Second Affiliated Hospital of Shaanxi University of Chinese Medicine, Xianyang, Shaanxi; b Graduate School of Beijing University of Chinese Medicine; c Department of Andrology; d The Fist Department of Neurology, Dongzhimen Hospital, Beijing University of Chinese Medicine, Beijing, China.

**Keywords:** acupuncture, prostate cancer, protocol, randomized controlled trial

## Abstract

**Introduction:**

Prostate cancer refers to an epithelial malignant tumor that occurs in the prostate area. In recent years, with the improvement of people's living standards, the incidence of prostate cancer has gradually increased, which has greatly affected people's life and health and quality of life. Acupuncture has its unique advantages in treating cancer pain. We will evaluate the efficacy and safety of acupuncture and moxibustion in the treatment of pain caused by prostate cancer using a clinical randomized parallel control method.

**Methods/design:**

This pragmatic randomized controlled trial will recruit 120 patients who are diagnosed with prostate cancer. Simple randomization to conventional drug treatment with a 1:1 allocation ratio will be used. Based on the patient's pain location and the primary lesion, the acupuncture needle insertion position was determined according to the principle of local selection of acupoints. All participants will continue to receive conventional drug treatment.

**Discussion:**

This trial may provide evidence regarding the clinical effectiveness, safety, and cost-effectiveness of acupuncture for pain caused by prostate cancer.

**Trial registration:**

ClinicalTrials.gov, ChiCTR2000029801, Registered on 14 February 2020.

## 1 Introduction

Prostate cancer refers to an epithelial malignant tumor that occurs in the prostate area. In recent years, with the improvement of people's living standards, the incidence of prostate cancer has gradually increased, which has greatly affected people's life and health and quality of life.^[1]^ Epidemiological surveys show that prostate cancer ranks second in the world (behind lung cancer) in male malignancies, accounting for 14% of all male cancer cases. The incidence of prostate cancer varies significantly worldwide, with the highest incidence being approximately 25 times the lowest incidence.^[2]^ Cancer pain is one of the most important symptoms in patients with advanced cancer. The incidence of this disease is about 70% to 90%, which greatly increases the physical and mental suffering of patients and affects the quality of life.^[3,4]^ Prostaglandins secreted by patients with prostate cancer will cause bone-absorbent nerve endings around the tumor to be extremely sensitive to the patient's painful stimuli, which can cause pain.

With the continuous progress of modern medicine, we are not only concerned about prolonging the life of cancer patients, we are more concerned about the impact of cancer on their own pain and quality of life. At present, modern medicine mainly uses the “three-step” analgesics recommended by WHO to treat cancer pain. Patients need long-term high-dose anesthetics for analgesia.^[5,6]^ American studies have found that long-term use of anesthetics often promote tumor angiogenesis, accelerate tumor growth, and promote the spread of cancer cells.^[7]^ In addition, clinical treatments have found that the use of this method often results in nausea, vomiting, dizziness, drowsiness, and other adverse reactions.

Acupuncture has its unique advantages in treating cancer pain. It can dredge the meridians, reconcile the blood, and improve the pain caused by Qi stagnation and blood stasis. Recent studies have shown that acupuncture can improve pain and quality of life in patients with prostate cancer.^[8,9]^ Therefore, in this study we will evaluate the efficacy and safety of acupuncture and moxibustion in the treatment of pain caused by prostate cancer using a clinical randomized parallel control method. We hope that the results of this study will provide sufficient evidence-based medical evidence for acupuncture treatment of pain caused by prostate cancer, and also provide better treatment methods for clinicians.

## 2 Methods/design

### 2.1 Study design and settings

**Figure 1:**
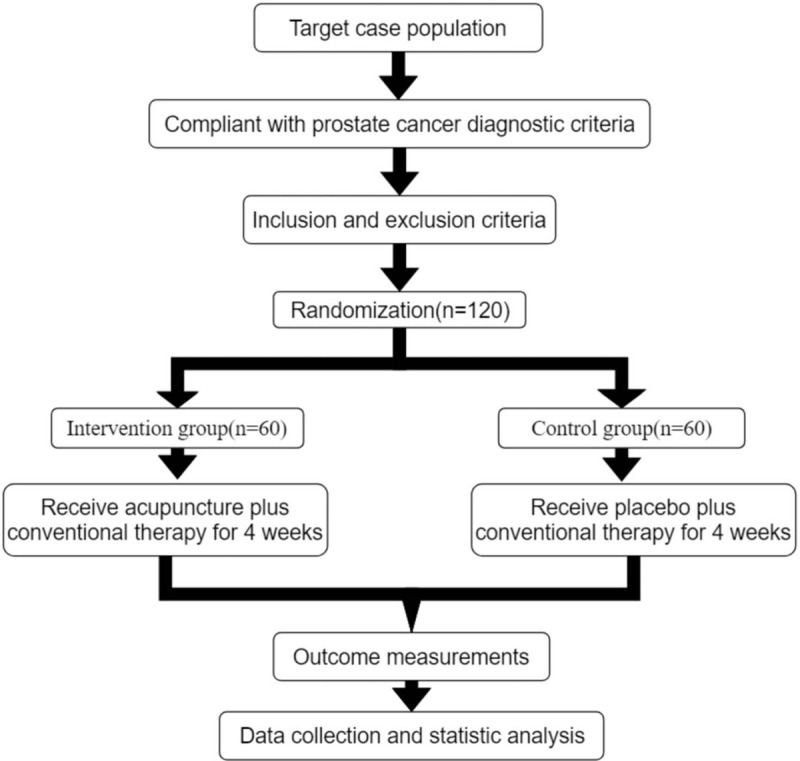
Study design flow chart.

A brief flowchart of the entire study is shown in Figure 1. We will perform a 2-group, randomized, single-blind, placebo-controlled, multi-center trial that will evaluate the efficacy and safety of acupuncture for patients with pain caused by prostate cancer. This study will use a completely random grouping and parallel control observation design method. We will ensure the balance of the baseline data of the two groups through a sufficient sample size and a completely randomized grouping method. This study will be approved by the Ethics Committee of The Second Affiliated Hospital of Shaanxi University of Chinese Medicine. We will not begin recruiting at other centers in the trial until local ethical approval has been obtained. This study is registered at http://www.chictr.org.cn/showproj.aspx?proj=49398 (ChiCTR2000029801). The protocol includes elements recommended in the Standard Protocol Items: Recommendations for Interventional Trials checklist (Additional file 1).

### 2.2 Setting and participants

This study will be conducted in China. Patients will be recruited from Urology/Andrology departments of The Second Affiliated Hospital of Shaanxi University of Chinese Medicine. We will enroll participants based on the following inclusion criteria:

1. Male patients diagnosed with prostate cancer will be included (refer to the diagnostic criteria established by the National Institutes of Health's consensus on expert diagnosis and treatment of prostate cancer pain syndrome);
2. The patient's age is 50 years or older;
3. Patients all had pain in the lumbosacral region, waist, chest and back, and pubic area to varying degrees; clinical manifestations were single / multiple site involvement.
4. Those who have signed informed consent;

Participants who meet the following exclusion criteria will not be enrolled:

1. Patients with severe organ dysfunction;
2. Patients unwilling to cooperate with the study;
3. Patients with mental cognitive dysfunction and whose severe mental illness has been detected;
4. Patients with a history of critical illness such as cirrhosis;
5. Patients with severe anemia;

### 2.3 Randomization, allocation concealment, and blinding

The web-based online randomization system to be used in this trial will be provided by an independent academic data management center at t he Second Affiliated Hospital of Shaanxi University of Chinese Medicine. The attending Physician will identify eligible patients according to the inclusion and exclusion criteria. Informed consent will be taken by the attending physician, and participants will be referred to a research coordinator who will randomly assign them to the intervention or control arm. The randomization list is kept by the biostatistician and research coordinator until the end of the study to ensure allocation concealment; therefore, the data analysts will be kept blinded to the allocation. The participants will be instructed not to disclose the allocation to the attending physician.

### 2.4 Interventions

Intervention group: Apply acupuncture for treatment. The method is as follows: Select a disposable acupuncture needle with a size of 25 mm × 0.25 mm. Based on the patient's pain location and the primary lesion, the acupuncture needle insertion position was determined according to the principle of local selection of acupoints. After local disinfection with 75% alcohol, acupuncture needles are inserted into the subcutaneous tissue. Adjust the number of acupunctures appropriately according to the patient's severity and self-tolerance. Patients with moderate or mild pain should be treated 4 days after stopping the use of analgesics, once a day, with 10 days as a course. After the treatment was completed, the recurrence and duration of pain symptoms were observed. Control group: At the same time as basic treatment, modern chemical analgesic drugs are used in accordance with the guiding principles of cancer three-step pain relief ladder therapy. Commonly used drugs:

1. Xilepan (manufacturer: Pfizer; batch number: BKl3CCEK164), 200 mg orally, once a day;
2. tramadol (manufacturer: Beijing Mengdi; batch number: Sinopharm standard 10083851), 10 mg, Orally, once a day;
3. Meishi Kangding (manufacturer: Beijing Mengdi; batch number: Sinopharm Standard 1405051), 10 mg orally, once a day.

### 2.5 Data collection

The study data collection process is outlined in Table 1.

**Table 1:**
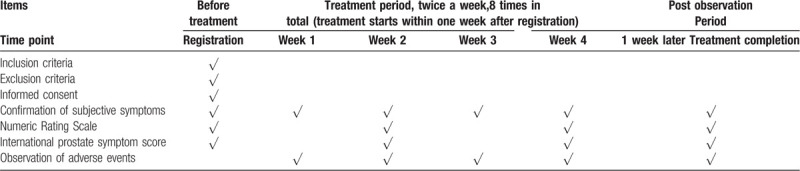
Treatment schedule and outcome measures.

### 2.6 Primary outcomes

To assess the pain level of patients with cancerous pain, we will use the Numeric Rating Scale, (NRS) pain standard. Specifically: 0 for painlessness; 1 to 3 for mild pain; 4 to 6 for moderate pain; 7 to 9 for severe pain; and 10 for severe pain. 0 to 10 points represent the patient's pain level. Significant effect: The patient's pain level is reduced by at least two or three levels, or the patient is painless; Effective: The patient's pain level is reduced by one level or the patient presents moderate or mild pain; Ineffective: The patient's pain does not have any relief, even worse trend.

### 2.7 Secondary outcomes

As for the secondary outcomes, we will apply the Quality of Life Score scale before and after treatment for patients with advanced prostate cancer. The scale is an assessment scale for the condition and quality of life of advanced, hormone-insensitive prostate cancer created by the University of California, USA. Including nine aspects of physical strength, pain, fatigue, appetite, family/marital relationship, mood, defecation, and general feeling. Each item is calculated with 100 points. The lower the score, the worse the situation. In addition, we will use the international prostate symptom score (IPSS) to evaluate prostate-related symptoms. 0 to 35 points according to the severity of the symptoms. Specific include whether there is endless urination, whether to go to bed at night to urinate, if you feel dysuria, whether you need to urinate within 2 h after urination, whether you can’t wait to urinate, or whether you feel the urine Thinning or whether to interrupt and start multiple times when urinating. 20 to 35 points are defined as severe symptoms; 8–19 points are defined as moderate symptoms; 0 to 7 points are defined as mild symptoms.

### 2.8 Statistical consideration

#### 2.8.1 Sample size

Based on the results of our previous study, we will use a 2-sided significance level of 5% and a power of 80%, the required number of patients in each group is 57.Considering dropout cases, we will enroll a total of 120 patients in this study.

#### 2.8.2 Analysis set

The Full Analysis Set will be used for all primary analysis. Per Protocol Set analysis will be performed for evaluating the sensitivity of results.

#### 2.8.3 Statistical analysis

Data management uses EXCEL software to build a database, double entry, check for outstanding values, and lock. Statistical analysis will be performed using SPSS 25.0 software for statistical analysis. The normality of the measurement data is tested. The data obeying the normal distribution is Student's t test, which is expressed by mean ± standard deviation. The data not obeying the normal distribution is rank sum test. And marginal homogeneity test; count data are expressed by rate and composition ratio, and comparison is performed by chi-square test; repeated measurement data are expressed by mean ± standard deviation, intra-group comparison is performed by analysis of variance of repeated measurement data, and inter-group comparison is by multivariate analysis of variance (MANOVA). *P *≤ .05 indicates that the difference is statistically significant.

### 2.9 Quality control and trial management

The management structure will comprise the principal investigator (PI), a trial management group, and a data monitoring committee. The trial management group will be responsible for conducting the trial and will meet monthly to discuss the trial progress. The PI will visit each collaborative hospital for face-to-face meetings and to share information to promote patient recruitment. The data monitoring committee will review safety and efficacy data. All data will be monitored every month through a central monitoring method. Additional monitoring may be performed at the discretion of the monitoring manager. The data monitoring committee have met once prior to the start of patient recruitment. At least twice per year, participating investigators, research assistants, and research nurses will be required to attend a training workshop on clinical research to ensure strict adherence to the study protocol and familiarity with the trial administration process. The data collected in this trial will comprise information recorded in case report forms and questionnaires. Data quality will be checked regularly by research assistants and overseen by monitors; all modifications will be marked on case report forms and data managers will recheck the data before they are officially logged. The database will be locked after all data have been cleaned. If participants withdraw from the trial during the study period, the reasons will be documented and the dropout rate will be statistically analyzed.

## 3 Discussion

Cancer pain is one of the most important symptoms in patients with advanced cancer. The incidence is about 70% to 90%, which greatly increases the physical and mental suffering of patients and affects the quality of life.^[10,11]^ According to statistics from the World Health Organization, there are about 10 million new cancer patients every year in the world, and 300 million to 1 billion cancer patients fail to receive timely and effective treatment.^[12]^ The trial results of many second-line hormones and chemotherapy methods are unsatisfactory and costly. In distant metastasis of prostate cancer, bone is the most common target organ, and bone metastasis occurs in 80% of prostate cancer patients.^[13]^ The lower lumbar spine is the most common site of bone metastases. Clinical manifestations often include chills, fatigue, bone pain, spinal nerve compression symptoms, osteoporosis, pathological fractures, and so on. Seriously reduced the quality of life of patients. At this time, the disease is in its advanced stage, and the research on palliative treatment is particularly important because of the lack of curative methods. The ultimate goal is to extend patient survival and improve quality of life.

Acupuncture analgesia has been widely used in tumor bone metastasis treatment and daily care because of its simple operation and no toxic and side effects such as oral drugs.^[14]^ Traditional acupuncture has analgesic effects by regulating neurotransmitter release, changes in hormone levels in the body, and reducing local edema between tissues. From the perspective of traditional Chinese medicine theory, acupuncture can dredge the meridians, reconcile the blood, and improve the pain caused by Qi stagnation and blood stasis.^[15]^ From the perspective of modern hospital research, the principle of acupuncture selection during acupuncture analgesia refers to the meridian theory. Through the body surface acupoints, it acts on internal organs and regulates the patient's neuroendocrine function, activating the body's pain modulation system to achieve analgesic effects. Recent studies have also shown that acupoint stimulation can release various mediators and opioid peptides. Together these endogenous substances form the body's “anti-pain system” and produce acupuncture analgesic effects. Therefore, in this study, we will evaluate the efficacy and safety of acupuncture in the treatment of pain caused by prostate cancer using a clinical randomized parallel control method. We hope that the results of this study will provide sufficient evidence-based medical evidence for acupuncture treatment of prostate cancer pain, and also provide better treatment methods for clinicians.

## Acknowledgments

The authors would like to thank all the trial participants. The authors are grateful for the support for this study: trial coordinating team, surgical staff, nurses, and research departments.

## Author contributions

YL, YYD, JSW, XDY, and RJL designed the study protocol and drafted the manuscript.HMS reviewed the study protocol and drafted the manuscript. JML is responsible for the statistical design and analysis as trial statistician. All authors carefully read and approved the final version of the manuscript.
